# Dynamic Edge Effects in Small Mammal Communities across a Conservation-Agricultural Interface in Swaziland

**DOI:** 10.1371/journal.pone.0074520

**Published:** 2013-09-09

**Authors:** Zachary M. Hurst, Robert A. McCleery, Bret A. Collier, Robert J. Fletcher, Nova J. Silvy, Peter J. Taylor, Ara Monadjem

**Affiliations:** 1 Department of Wildlife and Fisheries Sciences, Texas A&M University, College Station, Texas, United States of America; 2 Department of Wildlife Ecology and Conservation, University of Florida, Gainesville, Florida, United States of America; 3 Institute of Renewable Natural Resources, Texas A&M University, College Station, Texas, United States of America; 4 Department of Ecology & Resource Management, University of Venda, Thohoyandou, Limpopo, South Africa; 5 All Out Africa Research Unit, Department of Biological Sciences, University of Swaziland, Kwaluseni, Manzini, Swaziland; 6 Mammal Research Institute, Department of Zoology & Entomology, University of Pretoria, Pretoria, Gauteng, South Africa; University of Western Ontario, Canada

## Abstract

Across the planet, high-intensity farming has transformed native vegetation into monocultures, decreasing biodiversity on a landscape scale. Yet landscape-scale changes to biodiversity and community structure often emerge from processes operating at local scales. One common process that can explain changes in biodiversity and community structure is the creation of abrupt habitat edges, which, in turn, generate edge effects. Such effects, while incredibly common, can be highly variable across space and time; however, we currently lack a general analytical framework that can adequately capture such spatio-temporal variability. We extend previous approaches for estimating edge effects to a non-linear mixed modeling framework that captures such spatio-temporal heterogeneity and apply it to understand how agricultural land-uses alter wildlife communities. We trapped small mammals along a conservation-agriculture land-use interface extending 375 m into sugarcane plantations and conservation land-uses at three sites during dry and wet seasons in Swaziland, Africa. Sugarcane plantations had significant reductions in species richness and heterogeneity, and showed an increase in community similarity, suggesting a more homogenized small mammal community. Furthermore, our modeling framework identified strong variation in edge effects on communities across sites and seasons. Using small mammals as an indicator, intensive agricultural practices appear to create high-density communities of generalist species while isolating interior species in less than 225 m. These results illustrate how agricultural land-use can reduce diversity across the landscape and that effects can be masked or magnified, depending on local conditions. Taken together, our results emphasize the need to create or retain natural habitat features in agricultural mosaics.

## Introduction

With human population growth as its catalyst, agricultural production has become the dominant land-use on the planet, responsible for altering and endangering wildlife communities on a massive scale [1]–[3]. In particular, high-intensity farming has transformed native vegetation into monocultures thereby decreasing biodiversity on a landscape scale over the last several decades [4], [5]. This pattern has been especially evident over the last 40 years in Swaziland (52,233 ha of sugarcane cultivation in 2006/2007) [6] and eastern southern Africa (675,911 ha cultivation in 2009/2010) [7] where lowland savannahs have undergone continued conversion from native vegetation into sugarcane (*Saccharum* spp.) production [8], [9].

Within agricultural landscapes, native wildlife communities often utilize isolated patches of intact native vegetation interspersed throughout the croplands. One conservation concern about this landscape configuration is that conditions created at the interface (edge) of agricultural lands are likely to alter wildlife communities within natural areas, favoring generalists at the expense of specialists [10], [11]. Nonetheless, high productivity of agricultural lands may provide benefits such as increased food resources for wildlife and thus increased biodiversity at the landscape scale [12], [13]. Understanding the costs and benefits of habitat edges in agricultural landscapes remains an important issue in agricultural conservation [13].

While edge effects are incredibly common and are generally thought to help explain large-scale patterns in species distribution and community structure [14]–[16], edge effects are also considered to be highly dynamic and non-linear over space and time [17]–[20]. The responses of wildlife communities at adjoining land-uses often vary by species, season and microhabitat [21]–[24]. Furthermore, data on edge effects typically include spatial autocorrelation [14], which causes problems in making strong inferences on edge effects. These dynamic and autocorrelated effects have proven to be a major challenge for understanding edge effects and extrapolating these effects to landscape-scale patterns, in part because we currently lack an analytical framework for addressing this complex problem.

Our objectives were to: 1) evaluate changes in community structure between and across conservation and agriculture land-uses prevalent in much of south-eastern Africa, using eastern Swaziland as a model; and 2) extend existing non-linear models for edge effects [19] to account for dynamic spatio-temporal variation and problems of spatial autocorrelation in edge effect data [25]. We focused on small mammal communities because they play an important role in many ecosystems as herbivores, seed predators, and prey species and can be strong indicators of ecosystem health [26]–[29]. Small mammals appear to be altered by high-intensity agricultural [30], [31], but information on their community composition across agriculture and conservation land-uses is limited [32], [33] . We expected that small mammal communities would be less diverse and more homogeneous in agricultural lands than in conservation land-uses because of the simplified vegetative structure [4]. Furthermore, we expected changes in diversity would be site-and season-dependent. Our conservation sites varied in the quality, quantity and structure of vegetation, which should create site-level responses [28], [34]. Additionally, water resources from irrigated sugarcane should mitigate the normal seasonal fluctuations in small mammal communities [12], [28], [34].

## Materials and Methods

### Study Areas

**Figure 1:**
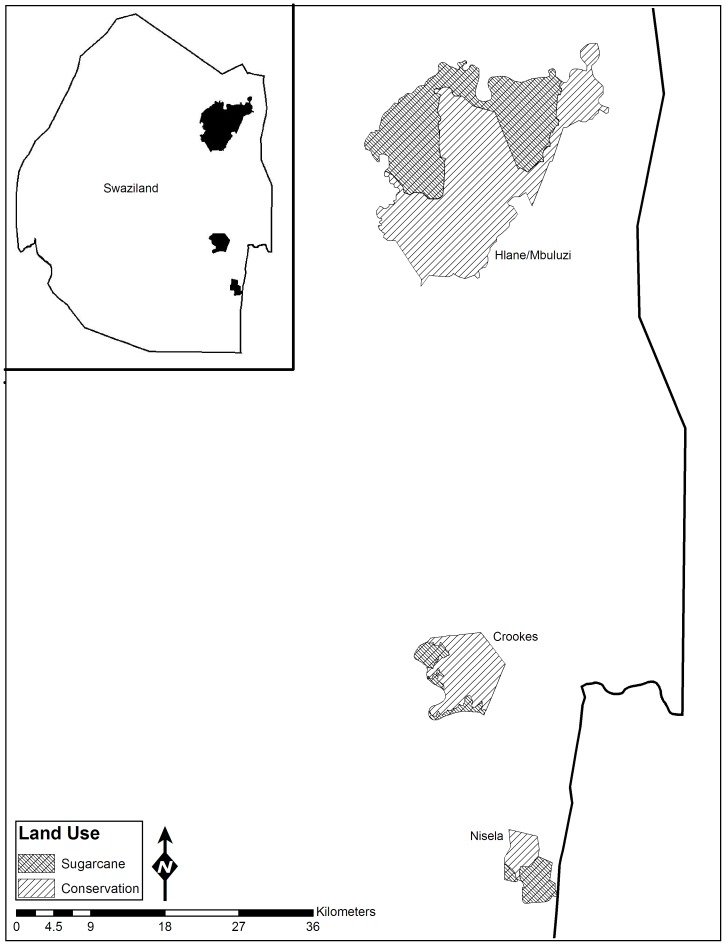
Map of the 3 study sites used to examine the effects of intensive agriculture on small mammal communities in the lowveld region of Swaziland. Inset: map of Swaziland showing the locations of the 3 sites.

We conducted our research in the lowveld of Swaziland between the northern Drakensburg Escarpment and the Lubombo Mountains (Figure 1). The lowveld lies in the eastern half of the country and is the lowest, warmest, and driest region. The vegetation of the region is characterized as lowveld savannah, with three distinct broad-scale vegetation types: *Acacia* savannah, broadleaved woodland, and riverine forest [22]. Swaziland has a subtropical climate, and exhibits distinct wet (October–March) and dry (April–September) seasons at approximately the same time each year (± one month), with 75% and 25% of rains falling during these respective seasons [35]. Annual precipitation ranges between 550–725 mm decreasing on a north-south gradient [36]. Within our Swaziland lowveld study region, all major sugarcane plantations adjoin *de facto* conservation areas, or lands managed for wildlife conservation, wildlife viewing, and sustainable grazing [37]. The juxtaposition of sugarcane with these conservation areas could potentially reduce the integrity and conservation value of lowveld’s conservation areas.

We conducted our research on three sites--Hlane-Mbuluzi, Crookes, and Nisela Farms--where conservation lands directly adjoined large-scale sugarcane plantations. Hlane-Mbuluzi included lands administered by Hlane Royal National Park, Mbuluzi Game Reserve, Tongaat-Hulett Sugar (Tabankulu Estate), and Royal Swazi Sugar Corporation (Simunye and Mhlume Estates). Conservation lands (Hlane Royal National Park and Mbuluzi Game Reserve) at Hlane-Mbuluzi were managed explicitly for wildlife conservation. Hlane-Mbuluzi did not allow cattle grazing and conducted land management activities such as prescribed burning. Adjacent sugarcane was irrigated and had both dirt and graveled access roads and 3 m high fence separating conservation lands and sugarcane restricting movements of medium- to large-sized mammals [38]. Crookes included lands managed by Crookes Brothers Plantation and Bar J Cattle Ranch. Conservation lands fell within the Big Bend Conservancy and were managed for wildlife conservation. Agriculture and conservation lands were separated by dirt access roads, 1.5 m barbed wire fence and 1 m wide irrigation canals. Additionally, there was an abrupt change in substrate with sugarcane cultivation ending as soils became rockier. Nisela was overseen by Nisela Farms with conservation lands managed for wildlife viewing, conservation, and grazing. Agricultural and conservation lands were separated by access roads, a railroad track section, and an electrified 3 m fence. Nisela experiences slightly lower rainfall than the other two sites and had lower canopies and less ground cover than Hlane-Mbuluzi or Crookes [39].

### Sampling Design

We used ground-truthed aerial photographs, Landsat images and GIS (ArcGIS 9.3, ESRI, Redlands, California), to identify the conservation-agriculture edge and generate sampling locations. We randomly (without replacement) generated 4 locations along the edges of each of the three study sites. In an effort to ensure spatial independence among locations, we did not allow random points to be placed within 300 m of another location, which is greater than the home range of the most widespread Muridae rodent in Swaziland [40], [41]. At each location, we placed a 750 m transect running perpendicular to the edge (0 m), extending 375 m into each land-use type. On each transect we placed traplines parallel to the edge at 0 m, and then 75, 150, 225, and 375 m into each land-use for a total of 9 traplines. Traplines consisted of 20 Sherman live traps set perpendicular to the transect and spaced 10 m apart for a total of 180 traps on each transect. Our design was expected to yield high levels of area surveyed per trap, and the relatively close spacing of traps ensured adequate sampling for species richness [42], [43]. We placed traps within 2 m of the assigned point in vegetation that would increase potential for capture and reduce weather exposure.

We surveyed each site once per season (dry  =  May–September, wet  =  October–March). During each sampling period, we trapped we trapped all traplines on a transect over four consecutive nights [44]. We started trapping in the dry season on July 5, 2008 and ended on October 8, 2008. We began trapping for the wet season after two weeks of measurable rain (>11 cm) on October 28^th^ and continued until January 6, 2009. We trapped site in the same order during each season.

We baited traps with a combination of oats and peanut butter, opened our traps in the morning and checked them each morning for four subsequent days. For each captured individual, we recorded species, age, sex, and reproductive condition [45], [46]. We collected additional information of body length, hind foot length, and mass [46]. We gave each individual ≥15 g a unique ear tag identifier (1005-1, National band Co., Newport, Kentucky, USA), smaller individuals and *Mus minutoides* were given ear punches (INS500075-5, Kent Scientific, Torrington, Connecticut, USA).

All captured species of shrews ( *Crocidura* spp. and *Suncus* spp.) were collected and deposited in the Durban Natural Science Museum (South Africa) for identification. We also collected voucher specimens of each rodent species from each site and deposited them in the collections of the Durban Natural Science Museum. All capture protocols and data collection followed guidelines outlined by the American Society of Mammalogists [47] and were authorized under Texas A&M University Animal Use Protocol (permit number 2008-98). Prior to sampling, we received permission for research from all land managers and a specimen export letter from the Swaziland National Trust Commission.

### Data Analysis

We calculated community metrics using minimum number known alive (MNA) estimates from each trapline (by site and season) [48] and used these metrics to evaluate small mammal community responses to conservation lands, agricultural lands and across the land-use interface (375 m into conservation – 375 into sugarcane). Despite being an index [49], we used MNA because low individual species capture rates limited our using more complex population estimation methods [50] across species and traplines.

Changes in community structure are not easy to encapsulate; therefore, we examined a set of complementary metrics, which included estimating dissimilarity of sampling units and measures of species richness and heterogeneity [48]. First, to examine changes in composition and patterns of dissimilarity in small mammal communities across land-uses and seasons we calculated a Bray-Curtis matrix for each distance (traplines pooled) along our gradient, for each site and season [51]–[53]. We calculated the Bray-Curtis similarity matrix using square root transformation of our capture data, which increased relative weight of less abundant species while maintaining variability in species abundances [54]. To understand differences in composition and patterns of dissimilarity between the land-uses and seasons we graphically displayed the similarity matrix using multi-dimensional scaling (MDS). To display the data using MDS we used 50 restarts to avoid errors from local minima [54], [55].

To further understand how small mammal communities changed across competing land-uses we estimated species richness (*S*; the number of species within a site), and a metric of heterogeneity using an inverse Simpson index (*D*; index combines *S* and evenness) at each trapline, on each transect [48]. To determine the influence of land-use, season and site for *S* and *D* we fitted generalized-linear mixed models. Each metric was modeled as a function of land-use, season and site, with transect as random effect using IBM SPSS Statistics (v.20). We examined the *F* statistics, degrees of freedom (*DF*) and p-value of each parameter to determine its importance in explaining variation in the metric.

**Figure 2:**
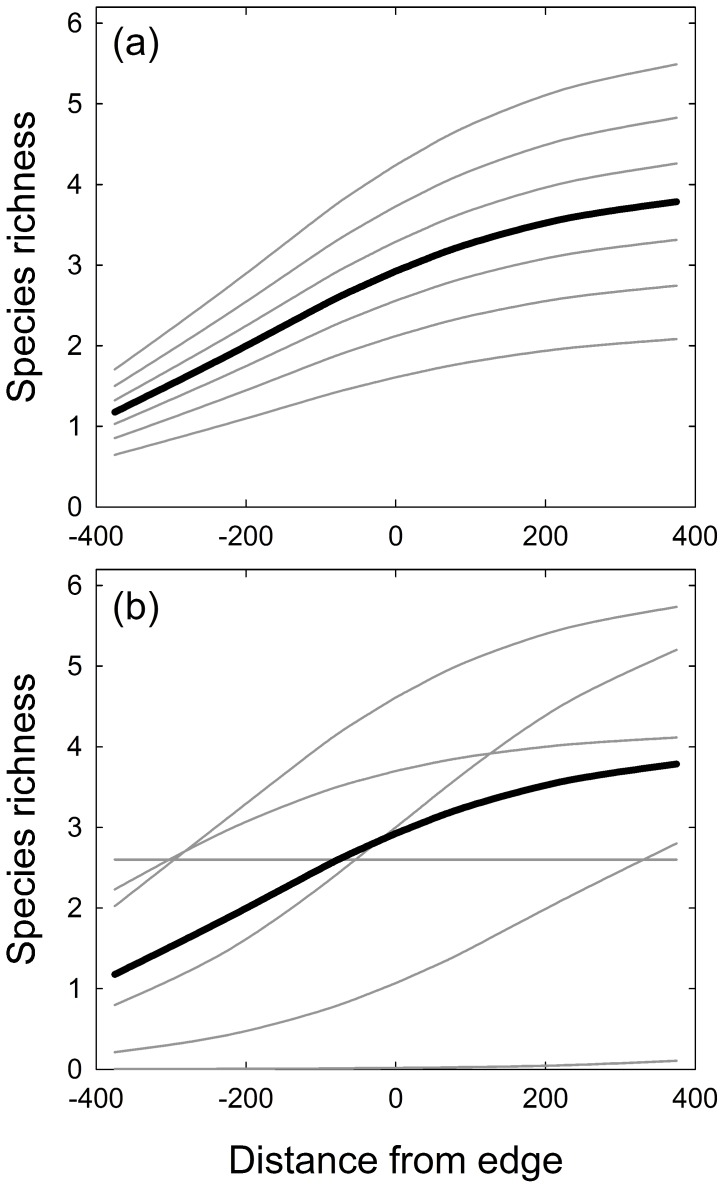
Hypothetical examples of logistic models to illustrate how they can quantify dynamic effects across space and/or time. Panel a represents a random-intercept (equation 1) and panel b represents a random coefficient (equation 2). For each model, the marginal (black line) and conditional (grey lines) predictions are shown. In these two examples, the fixed effects are the same and thus the marginal predictions are the same. The difference lies in the incorporation of conditional random effects, which allows for different forms of responses to occur across space or time.



Finally, we extended the non-linear models considered by Ewers and Didham [19] to account for hierarchical sampling designs, common in edge investigations, and the potential for edge effects to be dynamic over space and time. We fit a suite of non-linear mixed models to measures of *S* and *D* for each season to understand how diversity changed within and across land-uses. Like Ewers and Didham [19], we fit data to null (mean only), linear, power and logistic models. However, we added transect (n = 12) as a random variable to each model to account for spatial autocorrelation among traplines within transects and to better account for spatial variation in small communities across the edge among sites. To do so, we extended the models developed by Ewers and Didham [19] to allow for random intercepts (an additive effect of transect emphasizing different magnitudes of edge effects among transects) and random coefficients (allowing for different magnitude and extent of edge effects among transects; Figure 2). This extension is beneficial for three reasons. First, it properly accounts for hierarchical sampling designs (samples within transects, within sites), which is the common design for assessing edge effects [25]. Second, using random intercept and random coefficient models allow for heterogeneity in both the magnitude and extent of edge effects across transects that may not be captured from observed covariates. Third, it allows for both marginal and conditional predictions of edge effects. The random intercept logistic model was defined as:

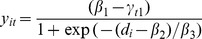
(1)Where *d_i_* is the distance of sample i from the edge, *β*_1-3_ are fixed parameters to be estimated that describe the shape of the logistic curve, and γ_t1_ is the random effect of transect t, N∼(0, σ_1_^2^), on the overall response (i.e., a random intercept). The random coefficients logistic model was defined as:
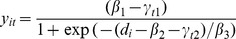
(2)


where γ_t2_ is the random effect of transect t, N∼(0, σ_2_^2^), on the distance effect.

We fit models to the data with SAS 9.2 (Cary, North Carolina) using the PROC NLMIXED for fitting non-linear mixed models. We defined the best fitting and most parsimonious model for each set of models (α*S* and β*S*, and α*D* for both seasons) as the model with the lowest Akaike’s Information Criterion corrected for small sample size (AICc). We graphically displayed the predicted fit and 95% CI of the best models (best linear unbiased predictors) along with the estimates for each transect (random effects; best linear unbiased estimates) across the land-uses for each season.

## Results

We trapped during the dry season from 5 July–13 October 2008 and wet season from 28 October 2008–10 January 2009, for 17,280 trap nights (8,640 per season). Our study areas received approximately 19% and 81% of the rainfall recorded during the dry and wet trapping seasons respectively (dry season trapping  =  57.9 mm, wet season trapping  =  221.7 mm). Over the duration of the study we captured 1,613 unique individuals representing 10 species (Table 1). All of the species were present 375 m in the conservation area, while only 4 species occurred 375 m into the sugarcane. The traplines furthest into the sugarcane (225 m and 375 m) were dominated by 3 species (*Mastomys natalensis, Mus minutoides, Lemniscomys rosalia*; Table 1) during both seasons.

**Table 1:** Number of individual small mammals captured (n) by species for traplines and their corresponding distance (m) across a conservation (+) - sugarcane (–) land-use gradient in the lowveld of Swaziland during the dry (May–September) and wet (October–March) seasons of 2008-2009.

Order	Species	(+375)	(+225)	(+150)	(+75)	(0)	(–75)	(–150)	(–225)	(–375)	Totals
											
*Macroscelidea*	*Elephantulus brachyrhynchus*	3	2	0	4	0	0	0	0	0	9
*Rodentia*	*Mastomys natalensis*	61	70	68	67	67	120	103	133	163	852
	*Mus minutoides*	22	22	23	30	35	41	31	28	25	257
	*Lemniscomys rosalia*	6	18	28	16	12	24	19	19	36	178
	*Aethomys inept us*	30	16	13	32	20	1	0	8	0	120
	*Steatomys pratensis*	13	13	13	16	4	0	0	0	0	59
	*Saccostomys campestris*	5	6	3	7	11	7	4	1	0	44
	*Gerbilliscus leucogaster*	4	9	8	2	6	5	3	0	0	37
*Soricomorpha*	*Crocidura hirta*	5	7	8	11	10	6	1	2	6	56
	*Suncus lixus*	1	0	0	0	0	0	0	0	0	1
	Total Individuals Captured	150	163	164	185	165	204	161	191	230	1613

**Figure 3:**
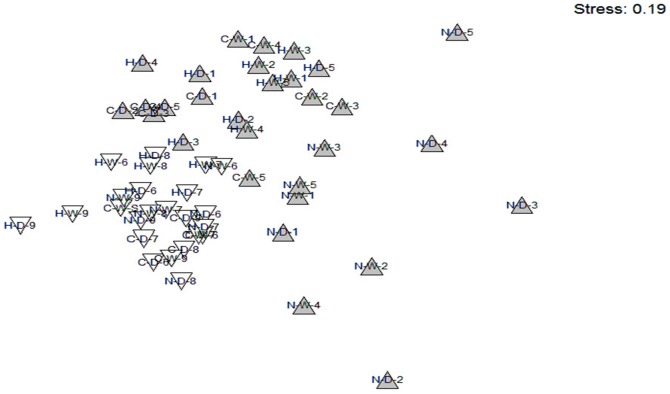
Non-metric multi-dimensional scaling plot showing the similarity of communities found within the conservation (shaded triangles) and sugarcane (white triangles) land-uses within the lowveld of Swaziland during wet and dry seasons captured between July 2008 and January 2009. Each community is identified by a 3 alpha-numeric symbols that represent site (H =  Hlane/Mubuluzi N = Nisela C =  Crookes) season (W = Wet D =  Dry) and trapline (1 to 5 = 375, 225, 150, 75 and 0 m into conservation areas 6–9 = 75, 150, 225, 375 into sugarcane).

Multi-dimensional scaling helped elucidate patterns graphically with distinct groupings formed by each land-use type; stress levels were acceptable (stress  =  0.19; [55]; Figure 3). We found the distribution of small mammal communities in the sugarcane was considerably more similar among transects than the communities in conservation areas, indicating less variation in the community structure of small mammals within the sugarcane.

Measures of *S* and *D* differed between seasons (*S*, *F* = 9.364, *df*  = 1, *p* = 0.003; *D*, *F* = 15.679, *df* = 1, *p* < 0.001), and land-uses (*S*, *F* = 6.736, *df*  = 1, *p* = 0.033; *D*, *F* = 6.908, *df*  = 1, *p* = 0.009), with greater species richness and heterogeneity in the wet season and in conservation areas (Table 2). However, these measures of community composition did not differ among the sites (*S*, *F* = 2.026, *df*  = 2, *p* = 0.135; *D*, *F* = 1.609, *df*  = 2, *p* = 0.203).

**Table 2:** Average species richness (*S*) and heterogeneity (inverse Simpson index, *D*) of small mammal species from Swaziland during of 2008/2009, by land-use (conservation, sugarcane), season (dry  =  May–September, wet  = October–March) and site (Hlane/Mbuluzi, Crookes, and Nisela).

Category		*S*	*D*
Land-use	Conservation	2.59 [2.14–3.04]	2.08 [1.80–2.35]
	Sugarcane	2.20 [1.74–2.66]	1.73 [1.45–2.02]
Season	Wet	2.67 [2.22–3.13]	2.16 [1.88–2.42]
	Dry	2.12 [1.66–2.57]	1.65 [1.37– 1.93]
Site	Hlane/Mbuluzi	2.66 [1.93–3.39]	2.07 [1.63–2.49]
	Crookes	2.62 [1.89–3.36]	2.06 [1.64–2.49]
	Nisela	1.91 [1.18–2.64]	1.59 [1.16–2.01]

Brackets show 95% CIs.

**Figure 4:**
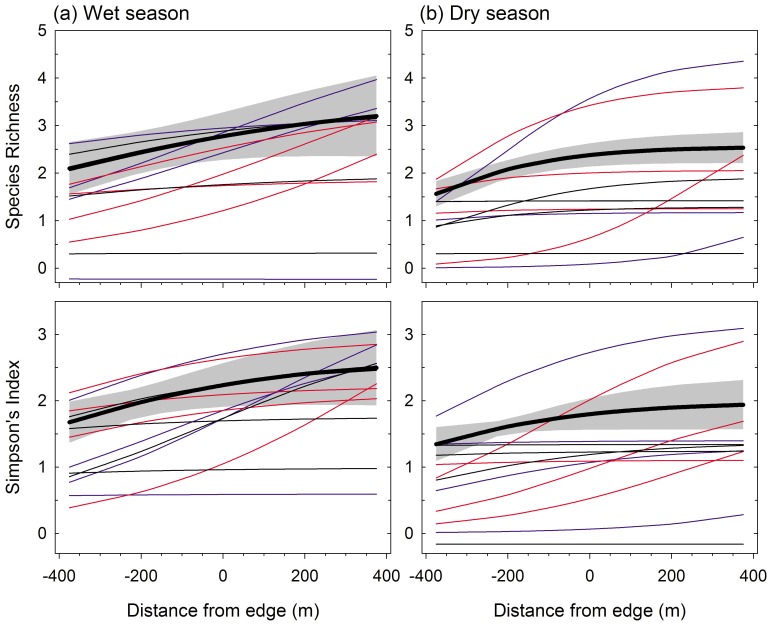
Marginal and conditional estimates of species richness and heterogeneity (inverse Simpson’s index) from best fitting models of small mammal community across a conservation/agriculture land-use interface in Swaziland during (a) wet (October–March) and (b) dry (May–September) seasons of 2008–2009. Black lines with shaded gray region represent the overall (marginal) model predictions and 95% CI of the best models. Blue, red and black lines represent conditional predictions for transects at Hlane/Mbuluzi, Crookes, and Nisela, respectively.

Examining the patterns of small mammal community change across the agricultural-conservation interface we found the random coefficient logistic model was the most parsimonious model for describing both *S* and *D* during both seasons (Table 3). The measures of diversity increased from the sugarcane into the conservation areas but the rates of change were more punctuated during the wet season (Figure 4). Conditional estimates of both *D* and *S* varied not only in magnitude but also in the extent of edge effects across the land-uses and did not appear to cluster by site (Figure 4). Note that we calculated second derivatives of equations 1and 2 in an attempt to quantify the extent of edge effects [19]; however, the inflection points were generally shallow and did not provide adequate quantification of the distance at which edge effects occur in this system.

**Table 3:** Compassion of model parsimony using Akaike’s Information Criterion corrected for small sample size (AICc), change in AICc from the best model (ΔAICC) and the number of parameters (*K*) for species richness (*S*) and heterogeneity (inverse Simpson index, *D*) of small mammals communities across a conservation/agriculture land-use interface in Swaziland during wet (October–March) and dry (May–September) seasons of 2008-9.

			*S*		*D*	
Season	Model	K	AICC	Δ AICC	AICC	Δ AICC
Wet						
	Null	3	379.1	9.3	320.7	12.0
	Linear	4	376.4	6.6	316.3	7.6
	power	4	373.1	3.3	313	4.3
	Logistic 1 (intercept)	5	375.1	5.3	314.9	6.2
	Logistic 2 (coefficient)	7	369.8	**0.0**	308.7	**0.0**
Dry						
	Null	3	384.8	30.9	304.4	25.7
	Linear	4	385.9	32.0	306.0	27.3
	power	4	381.7	27.8	303.6	24.9
	Logistic 1 (intercept)	5	381.3	27.4	304.1	25.4
	Logistic 2 (coefficient)	7	353.9	**0.0**	278.7	**0.0**

## Discussion

We examined small mammal communities across agricultural-conservation interfaces in southern Africa and found considerable variation in measures of diversity between land-uses, seasons and patterns of change across the land-use interface. Sugarcane plantations showed significant reductions in species richness and heterogeneity, and greater similarity in community composition when compared with conservation areas. The sugarcane land-use was dominated by a homogenized community of three species: two generalist species with omnivorous diets (*Mastomys natalensis, Mus minutoides*) and a single herbivore (*Lemniscomys rosalia*) [34], [46]. These results correspond with research showing that intensive agriculture has minimal impact on generalist and herbivorous species while reducing the populations of more specialized species [56]–[59].

We did not see the increases in diversity around the interface of the land-uses that are commonly found in other studies [60]. Edge-related increases in diversity occur when species can utilize resources from multiple habitats or when adjoining land-uses create a unique set of resource or conditions [18], [61], [62]. However, these conditions are less likely to occur around abrupt edges, such as when high-intensity agricultural lands adjoin natural areas [62]–[65].

There was less variation in small mammal richness and heterogeneity across the land-use interfaces during the dry season (Figure 4). Trapping data and multi-dimensional scaling analysis indicated the same species were found in sugarcane throughout the year, while more specialized species (e.g. *Aethomys ineptus, Steatomys pratensis, Elephantulus brachyrhynchus, Gerbilliscus leucogaster*), were generally limited to conservation areas and more common during the wet season (Table 1, Figure 3). Some of these specialist species enter torpor and burrow during the dry season, and most limit their dispersal and recruitment of young to the wet season, when their activity increases[46]. These traits might reduce detection and densities of specialist species and subsequently explain the moderated variation in diversity across land-uses and the overall drop in diversity during the dry season (Figure 4).

The three conservation areas under study differed in grazing pressure, fire management, vegetative structure and soils; yet, there were no clear differences in diversity among the three sites. In general, measures of diversity (richness and heterogeneity) showed a similar pattern of increasing towards the edge (from the sugarcane), and tending to plateau within 200 m into the conservation areas (Figure 4). However, variation in these patterns were most evident at the transect level rather than the site level. We can only speculate as to the causes of variation in small mammal community response, but other studies have suggested differences in vegetative structure, microclimate, soils or other factors that might influence small mammal diversity at fine scales around edges [64], [66], [67].

By extending Ewers and Didham’s [19] non-linear modeling, we were able to describe the dynamic response of communities around the interfaces of these land-uses more accurately. We eliminated a common problem of spatial autocorrelation for many edge effect studies (dependency among trap sites within transects) by accounting for transects as a random variable. This addition coupled with the inclusion of models that accounted for variation in the pattern, magnitude and extent of localized edge effects (i.e. models with random coefficient and intercept variables) allowed for a more realistic representation of community level changes across land-uses. Our approach allowed for the inclusion of varied responses at a fine scale while allowing us to understanding the broader patterns of community change across the landscape. This mixed modeling framework could be further extended to account for other dynamic variation in space and time via the inclusion of other random effects and explicitly modeling the variance-covariance matrix of random effects.

### Conservation Implications

In our study, sugarcane plantations contained no unique small mammal species and diversity decreased with distance into the sugarcane. Additionally, we did not observe an increase in species diversity at the conservation-sugarcane edge. The extensive sugarcane plantations that abut conservation areas throughout Swaziland and in many other parts of south-eastern Africa appeared to have no measurable positive effect on small mammal diversity.

Globally, the vast areas of the earth’s surface dedicated to agriculture are increasingly farmed using high-intensity practices [4], [5], like those used to produce sugarcane in Swaziland and southern Africa. Using small mammals as indicators, intensive agricultural practices may be altering vertebrate community structure on a massive scale, creating high density communities of generalist species while isolating specialist species to localized areas of undisturbed habitats. In southern Africa, this pattern of isolation is further exacerbated because alternative land-uses such as intensive grazing have been shown to be equally detrimental to small mammal communities and interior species in particular [68], [69]. The isolation of small mammals in patches of conservation habitat may result in reduced population viability, gene flow and increasing susceptibility to stochastic events [70]–[72], in turn, negatively affecting the wildlife communities persisting in conservation areas and other patches of native vegetation.

Conservation efforts cannot be focused solely on isolated areas of pristine or intact vegetation. Broader approaches which view conservation in natural areas in conjunction with agricultural development are necessary to promote conservation within the agricultural mosaic landscape. Agricultural landscapes can be managed for mosaics of different patches that maximize cover and connectivity and retain natural habitat features, all of which help mitigate stressors on wildlife communities [12], [73], [74].

Although our research does not directly address these problems, the extension of Ewers and Didham’s (2006) methodology for examining edge effects will allow researchers and managers to more clearly identify local differences in edge effects across their study sites. Researchers can use this information to focus on investigations of the mechanisms driving community diversity on fine scales, while managers can use this methodology to identifying areas and characteristics associated with high diversity and areas in need of management or restoration.
